# Selection and Application of ssDNA Aptamers to Detect Active TB from Sputum Samples

**DOI:** 10.1371/journal.pone.0046862

**Published:** 2012-10-04

**Authors:** Lia S. Rotherham, Charlotte Maserumule, Keertan Dheda, Jacques Theron, Makobetsa Khati

**Affiliations:** 1 Emerging Health Technologies Platform, Council for Scientific and Industrial Research, Biosciences Unit, Pretoria, South Africa; 2 Department of Microbiology and Plant Pathology, University of Pretoria, Pretoria, South Africa; 3 Department of Medicine, Groote Schuur Hospital and University of Cape Town, Cape Town, South Africa; National Institute of Infectious Diseases, Japan

## Abstract

**Background:**

Despite the enormous global burden of tuberculosis (TB), conventional approaches to diagnosis continue to rely on tests that have major drawbacks. The improvement of TB diagnostics relies, not only on good biomarkers, but also upon accurate detection methodologies. The 10-kDa culture filtrate protein (CFP-10) and the 6-kDa early secreted antigen target (ESAT-6) are potent T-cell antigens that are recognised by over 70% of TB patients. Aptamers, a novel sensitive and specific class of detection molecules, has hitherto, not been raised to these relatively TB-specific antigens.

**Methods:**

DNA aptamers that bind to the CFP-10.ESAT-6 heterodimer were isolated. To assess their affinity and specificity to the heterodimer, aptamers were screened using an enzyme-linked oligonucleotide assay (ELONA). One suitable aptamer was evaluated by ELONA using sputum samples obtained from 20 TB patients and 48 control patients (those with latent TB infection, symptomatic non TB patients, and healthy laboratory volunteers). Culture positivity for *Mycobacterium tuberculosis* (Mtb) served as the reference standard. Accuracy and cut-points were evaluated using ROC curve analysis.

**Results:**

Twenty-four out of the 66 aptamers that were isolated bound significantly (p<0.05) to the CFP-10.ESAT-6 heterodimer and six were further evaluated. Their dissociation constant (K_D_) values were in the nanomolar range. One aptamer, designated CSIR 2.11, was evaluated using sputum samples. CSIR 2.11 had sensitivity and specificity of 100% and 68.75% using Youden’s index and 35% and 95%, respectively, using a rule-in cut-point.

**Conclusion:**

This preliminary proof-of-concept study suggests that a diagnosis of active TB using anti-CFP-10.ESAT-6 aptamers applied to human sputum samples is feasible.

## Introduction

TB is a major fatal infectious disease. The current World Health Organization (WHO) figures estimate a worldwide TB incidence of 8.8 million per year and 1.45 million deaths annually [1]. This statistic is compounded by the emergence of drug-resistant strains of TB [2], [3], [4] and co-infections with human immunodeficiency virus (HIV) [5].

Timely diagnosis of active pulmonary TB cases is important for the control of the disease. Despite the enormous burden of TB, conventional approaches to diagnosis used today rely on tests that have major drawbacks. The current gold standard for diagnosing TB is smear microscopy and culture. The major drawback of sputum smear microscopy is its poor sensitivity − estimated at 50% [6], [7]. The culture method is the most sensitive, however, it is more expensive than microscopy, requires up to eight weeks for the isolation of *Mycobacterium tuberculosis* (Mtb), and requires a high standard of technical competence [8]. Other methods available for diagnosing active TB include the lipoarabinomannan (LAM) antigen-detection assay [9], [10] and, more recently, a fully automated polymerase chain reaction (PCR)-based molecular test called GeneXpert®. The GeneXpert® has advantages in that it is rapid and little training is required. However, the drawback is that the test is currently expensive and not a point-of-care tool. Furthermore, the sensitivity of the GeneXpert® in the South African setting was found to be suboptimal in smear-negative, HIV-infected patients [11].

Accurate diagnosis of TB requires reliable biomarkers as targets of detection. Two Mtb proteins that have attracted attention as desirable targets for new TB drugs and diagnostics are CFP-10 and ESAT-6 [12], [13], [14], [15]. The CFP-10 protein, encoded by the Rv3874 (*esxB*) gene, and the ESAT-6 protein, encoded by the Rv3875 (*esxA*) gene, can interact *in vitro* to form a tight 1∶1 heterodimer [16], [17]. These two proteins are potent T-cell antigens recognised by over 70% of tuberculosis patients and are thus good TB biomarkers [18], [19], [20]. Moreover, CFP-10 and ESAT-6 are not present in many nontuberculous mycobacteria and in the *Mycobacterium bovis* Bacillus Calmette-Guérin (BCG) vaccine [5], [18], [19]. A recent study reported on the use of antibodies to detect the two proteins in clinical specimens with a sensitivity of 81.6% and 95.4% for CFP-10 and ESAT-6, respectively, and a specificity of 92.2% and 100% for the two proteins, respectively [20]. This data bodes well for a diagnostic tool that detects the antigens in clinical specimens. However, the use of antibodies as detection reagents has some limitations, which can be circumvented by aptamers.

Aptamers, by virtue of their high specificity and high sensitivity, could serve as tools for the early and specific detection of active TB and meet the ASSURED (Affordable, Sensitive, Specific, User-friendly, Rapid and robust, Equipment-free, and Deliverable to the end user) diagnostic guidelines recommended by WHO for developing countries [21]. The isolation of aptamers, with the capacity to recognise virtually any class of target molecule with high affinity and specificity, has been made possible by the development of the systematic evolution of ligands by exponential enrichment (SELEX) process [22], [23], [24]. Since aptamers can be produced using chemical synthesis or by PCR, the cost of producing aptamers is 10–50 times less than those for producing antibodies [25]. Aptamers have been raised against a wide variety of targets, from small human molecules and viral proteins to whole microorganisms [26].

Single-stranded DNA (ssDNA) aptamers are usually used for diagnostics due to the greater inherent stability of DNA and low cost of production compared to RNA aptamers. Examples include aptamers that detect thrombin, adenosine and cocaine [27], [28], [29], [30], [31]. Aptamers have also been raised against (1–3)-b-D-glucans, which were the first aptamers developed for the detection of a biotoxin in environmental respiratory diseases [25]. TB-specific aptamers include ssDNA aptamers against Mtb polyphosphate kinase 2 (PPK2) [32], as well as aptamers against the whole Mtb bacterium [33]. Furthermore, aptamers against MPT64 were the first ones raised against Mtb [34], but this antigen lacks the sensitivity of more potent antigens like ESAT-6 and CFP-10. To date, however, aptamers have not been raised against the immuno-dominant antigens CFP-10 and ESAT-6, or the CFP-10.ESAT-6 heterodimer.

The aim of this study was therefore to isolate ssDNA aptamers against the CFP-10.ESAT-6 heterodimer and test the ability of these aptamers to detect Mtb in sputum samples from patients with culture-proven tuberculosis.

## Materials and Methods

### Ethics Statement

Approval for the use of sputum samples for this study was obtained from the University of Cape Town, Health Science Faculty Research Ethics Committee (Ethics number: REC REF: 421/2006). Written consent was received from patients whose sputum is used in the study.

### Expression and Purification of Recombinant CFP-10 and ESAT-6 Proteins

*E. coli* BL21(DE3) cells (Novagen) were transformed with the T7 promoter-based expression plasmids pMRLB46 or pMRLB7 for expression of full-length histidine (His)-tagged CFP-10 and ESAT-6 proteins, respectively. Expression of the recombinant proteins was induced in mid-exponential phase cultures, by addition of isopropyl-β-D-galactopyranoside (IPTG). For cultures expressing CFP-10, the cells were harvested 4 h after induction, whereas for ESAT-6, the induced cultures were incubated overnight at 25°C before the cells were harvested. The respective cell pellets were suspended in 10 ml of Buffer A (6 M urea, 25 mM Tris-HCl, 200 mM NaCl, 10 mM imidazole, pH 7.4). The cells were disrupted by sonication (Bandelin Sonoplus HD2070), after which the lysate was clarified by centrifugation (14,000 rpm for 15 min at 4°C). For CFP-10, the supernatant (cytosol fraction) was loaded onto a nickel-nitrilotriacetic acid (Ni-NTA) column, which had been pre-equilibrated with Buffer A. The urea was removed by six washes with 10 ml of Buffer A, in which the urea concentration was decreased from 6 M to 1 M, followed by two washes with 20 ml of Buffer B (25 mM Tris-HCl, 200 mM NaCl, 10 mM imidazole, pH 7.4). Finally, CFP-10 was eluted in a single step with 10 ml of Buffer B containing 300 mM imidazole. ESAT-6 was purified from the cytosol fraction using a protocol similar to that used for the purification of CFP-10, except that the Ni-NTA column was washed successively with 20 ml of Buffer B containing 40 mM and 50 mM imidiazole, respectively, before elution of the ESAT-6 protein in 300 mM imidazole. The purified CFP-10 and ESAT-6 proteins were resolved by 17% SDS-PAGE and electroblotted onto Hybond™-ECL nitrocellulose membranes (GE Healthcare) for immunoblot analysis using either an anti-CFP-10 polyclonal antibody (provided by Megan Lucas, Colorado State University) or an anti-ESAT-6 monoclonal antibody (Santa Cruz Biotechnology).

### Complex Formation of CFP-10 and ESAT-6

Equimolar amounts of CFP-10 and ESAT-6 were mixed in a phosphate buffer at room temperature, as described previously [17]. Complex formation of CFP-10 and ESAT-6 was confirmed by native PAGE analysis and surface plasmon resonance (SPR) using a BIAcore® 3000 instrument.

### Isolation of Aptamers against the CFP-10.ESAT-6 Heterodimer

A 90-mer ssDNA randomised at 49 nucleotide positions and flanked by constant regions was custom-synthesised by Integrated DNA Technologies (CA, USA). The constant regions allowed for primer annealing and PCR amplification. The primer sequences were 5′-GCCTGTTGTGAGCCTCCTAAC-3′ (forward primer) and 5′- GGGAGACAAGAATAAGCATG-3′ (reverse primer modified with either the T7 promoter region TAATACGACTCACTATA, or a phosphate at the 5′ end). The library used was 5′-GCCTGTTGTGAGCCTCCTAAC(N49)CATGCTTATTCTTGTCTCCC-3′. The selection of aptamers was done according to the SELEX protocol, as described previously [35]. Briefly, before the first round of selection, the ssDNA library was incubated with a nitrocellulose membrane to remove any membrane-binders. The ssDNA was refolded by initial denaturation at 95°C for 10 min, followed by immediate cooling on ice for 5 min and then incubated at room temperature for 5 min in HMCKN binding buffer (20 mM HEPES, 2 mM MgCl_2_, 2 mM CaCl_2_, 2 mM KCl and 150 mM NaCl, pH 7.4). This was then incubated with 1590 nM of CFP-10.ESAT-6 heterodimer for 1 hour at 37°C. The ssDNA-protein mixture was passed through a nitrocellulose membrane. Non-specifically bound ssDNA was removed with two washes of HMCKN binding buffer, whereas ssDNA bound to the heterodimer was recovered with a 7 M urea buffer and purified for use in the next round of selection.

After the first round of selection, PCR was used to amplify the selected ssDNA into double stranded DNA (dsDNA). All PCR procedures were carried out under the following conditions: 95°C for 3 min, 8–12 cycles at 95°C for 1 min, 54°C for 1 min and 72°C for 90 sec, followed by a final extension at 72°C for 8 min. After the PCR, two different methods were used to generate ssDNA from the dsDNA. The first method used the dsDNA as a template for *in vitro* transcription to obtain RNA. The RNA was treated with DNase I, purified and precipitated. It was then used as the template in an RT-PCR to obtain cDNA. The cDNA-RNA mixture was treated with a 1 M NaOH, 0.5 M EDTA buffer to hydrolyse the RNA. The cDNA was purified and used as template for the next round of selection (this method is referred to as ‘T7-based SELEX’). The second method used to generate ssDNA was by digestion of the dsDNA with lambda exonuclease; this was done by including a phosphate on the 5′ end of the reverse primer. The dsDNA was purified and treated with lambda exonuclease (New England Biolabs), resulting in the degradation of the reverse strand with the phosphate modification [36]. The resulting ssDNA was then purified and used as template for the next round of selection. This method is referred to as ‘exonuclease-based SELEX’.

To minimise the possibility of selecting aptamers that bind to the nitrocellulose partitioning membrane, the aptamer pool from the fifth T7-based and third exonuclease-based SELEX rounds were respectively subjected to a counter-selection against the nitrocellulose partitioning membrane. The pools recovered after counter-selections were put through final SELEX rounds (i.e. sixth – T7-based SELEX and fourth – exonuclease-based SELEX, respectively). In this last round, both pools of ssDNA were amplified with the phosphate-modified reverse primer and the resultant PCR products were cloned into the pGEM-T Easy vector (Promega). Nucleotide sequencing of each individual clone was performed by Inqaba Biotechnical Industries (Pretoria, South Africa) using universal pUC/M13 sequencing primers.

### Binding Assay of ssDNA Aptamers by ELONA

Unique aptamers identified by nucleotide sequencing were tested for their binding ability to the CFP-10.ESAT-6 heterodimer, as well as the individual monomers (CFP-10 and ESAT-6) using an ELONA [37]. Briefly, each ssDNA aptamer was prepared with a biotinylated forward primer using the exonuclease method as described above. Protein concentrations were determined by a Bicinchoninic acid protein assay (BCA) so that wells could be coated with equimolar amounts of the heterodimer or with the respective monomers. The 96-well microtitre plates were coated with 500 ng of the proteins in 100 µl of NaCO_3_ buffer overnight at 4°C. Following blocking with a 5% fat-free milk solution for 1 hour at 4°C, the wells were washed four times with TBS buffer. Biotinylated aptamers (500 nM) were added to each well and incubated for 2 hours at room temperature. The wells were washed four times with TBS buffer. Streptavidin-horseradish peroxidase conjugate (KPL) was diluted 1∶15 000 in TBS buffer, and 100 µl was applied to each well. The plates were incubated for 2 hours at 37°C and washed again as described above. Then, 50 µl of Turbo-3,3′,5,5′-tetramethylbenzidine (TMB, Pierce) was added to each well and incubated for 15 min at 37°C. The reaction was quenched by addition of 50 µl of 1 M H_2_SO_4_, and the protein-bound aptamer-streptavidin complexes were quantified by determining the absorbance at 450 nm using a MultiSkan Go plate reader (Thermo Scientific).

### Aptamer-antibody Competition Assay

The aptamer-antibody competition binding was performed using the ELONA method described above with minor modifications. An ESAT-6 monoclonal antibody (Santa Cruz Biotechnology) was coated onto a 96-well microtitre plate overnight, followed by blocking and binding of the heterodimer. The biotinylated aptamer was then added, followed by the HRP-conjugated streptavidin. The data obtained from the competition assay was compared to that from the affinity binding assay to identify differences in binding capacity of the aptamers in the presence of the antibody. Although the competition and binding assays were performed on different plates, the positive control, CFP-10.ESAT-6 heterodimer and one aptamer, were tested on both plates with similar results. Data from one plate was normalised to the control of that plate, and compared to data from the other plate.

### Determination of the Dissociation Constant (K_D_) of Individual ssDNA Aptamers

To determine the affinity of the ssDNA aptamers to CFP-10 kinetic studies were conducted using a BIAcore® 3000 instrument. All four flow cells on a CM5 chip (BIAcore, Separations Scientific) were activated with 1-ethyl-3-(3-dimethylaminopropyl) carbodiimide (EDC) and N-hydroxysuccinimide (NHS) (BIAcore, Separations Scientific). CFP-10 was coupled to three of the four flow cells and any remaining carboxy site on the chip surface was blocked using 1 M ethanolamine-HCl. The ssDNA aptamer (at concentrations 500 nM, 250 nM, 125 nM, 62 nM, and 31 nM) was injected over all activated flow cells at a flow rate of 10 µl/min for 5 min with a dissociation time of 10 min. This was repeated for each of the six selected ssDNA aptamers. The flow cell that did not have CFP-10 was used as the control to subtract non-specific binding. The evaluation was done using BiaEvaluation Software (BIAcore) to determine the K_D_ values for each aptamer.

### Determination of the Effect of Aptamer Folding on Target Binding

To assess the downstream application of one ssDNA aptamer, namely CSIR 2.11, as a potential detection reagent for TB, we determined whether or not the aptamer needed to be refolded to retain its functionality. One batch of CSIR 2.11 was folded as described earlier, and the other batch of CSIR 2.11 was used directly after thawing. An ELONA was performed with both batches of CSIR 2.11 to determine binding to the CFP-10 monomer.

### Specificity Testing

To determine specificity, all six aptamers were tested in an ELONA for binding to lysates of different bacteria found in the oral cavity, namely: *Pseudomonas aeruginosa*, *Streptococcus pyrogenes*, *Staphylococcus aureus*, *Mycobacterium smegmatis*, *Mycobacterium bovis* BCG, *Mycobacterium tuberculosis*, and *Corynebacterium xerosis.* Lysates were obtained by bead-beating 200 ml of each culture. A bicinchoninic acid (BCA) protein assay was used to determine the total protein concentration of the prepared lysates. An equal amount of total protein was then coated onto 96-well microtitre plates using the *Mycobacterium tuberculosis* lysate as the positive control.

### Antigen Detection in Sputum Samples

**Figure 1:**
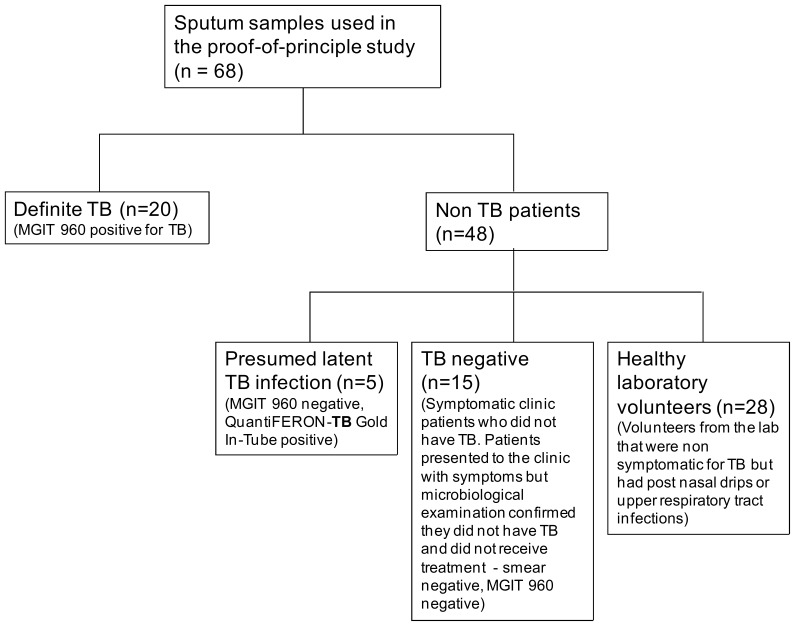
Study flow diagram. Study plan and patient categorisation of the 68 participants in active (definite) TB, latent TB, TB negative and healthy donors that were evaluated in the proof-of-principle study.

To determine the use of the ssDNA aptamers as detection reagents, one suitable ssDNA aptamer was tested in a small case controlled study. Forty sputum samples that have been well characterised were obtained from the Lung Infection and Immunity Unit, Groote Schuur Hospital and the University of Cape Town (South Africa), were used for the clinical study. A further fifteen apparently healthy volunteers working at the CSIR donated one to two sputum samples for testing (Figure 1). All sputum samples obtained were liquefied in a 0.1% dithiothreitol (DTT) solution. The sputum samples were bound to a 96-well microtitre plate in NaHCO_3_ buffer and left overnight at 4°C. An ELONA was performed to determine whether the aptamer was able to detect CFP-10 in sputum samples using CSIR 2.11. A specificity and sensitivity comparison, by means of an ELONA, between CSIR 2.11 and CSIR 2.21 was performed using a small subset of samples (28 sputum samples). CSIR 3.13, which is an aptamer selected from the same parental library but against an unrelated target was used as a negative control.

### Statistical Analysis

For binding and aptamer-antibody competition assays, each plate had an aptamer alone control, which was averaged, before subtracting the average from each well to eliminate background noise. Each aptamer or aptamer-antibody combination was tested in triplicate, in two separate experiments. The repeats were averaged and the standard deviation calculated. Statistical significance was calculated relative to the aptamer alone control using a two-tailed Student *t*-test. For the kinetic studies the BIAevaluation software calculates a Chi^2^ value for the dissociation constant. For the small case controlled study each sputum sample was tested in triplicate. The results were averaged and the standard deviation was calculated for each sputum sample. Each experiment was repeated twice to determine repeatability of the assay by calculating the coefficient of variance. The diagnostic performance of the assay was evaluated using ROC curve analysis (SigmaPlot 12.0, Statsoft, Inc.; GraphPad Prism 5.04, GraphPad Software, Inc.) and cut-points were determined based on Youden’s index, as well as the rule-in method.

## Results

### Protein Expression, Purification and Heterodimer Formation

The His-tagged CFP-10 protein was purified from the soluble fraction of induced cell lysates using nickel affinity chromatography. SDS-PAGE analysis showed the presence of CFP-10 without any visible contaminants (Figure S1A), and the purified protein was shown by immunoblot analysis to react with an anti-CFP-10 polyclonal antibody (Figure S1B). Typically, 2.4 mg of monomeric CFP-10 was obtained from 1 l of bacterial culture. In contrast to CFP-10, the ESAT-6 protein is expressed in *E. coli* predominantly as an insoluble protein and solubilisation of the protein requires exposure to a strong chaotropic agent such as guanidine-HCl or urea at concentrations from 6–8 M [15], [17]. Here, we adopted a strategy aimed at limiting *in vivo* aggregation of the recombinant protein by growing the IPTG-induced bacterial culture overnight at a reduced temperature (25°C). Using this approach, the ESAT-6 protein was localised to the soluble fraction of the induced cell lysate. This finding is in agreement with other reports regarding expression of different antigens of *M. tuberculosis*, which indicated that lowering of the growth temperature leads to enhanced solubility [15], [38]. Following purification, SDS-PAGE analysis of the eluate indicated that it contained the ESAT-6 protein (Figure S1C), which reacted specifically with an anti-ESAT-6 monoclonal antibody in immunoblot analysis (Figure S1D). The purification procedure yielded 1.3 mg of monomeric ESAT-6 from 4 l of bacterial culture.

To determine whether the recombinant CFP-10 and ESAT-6 proteins were capable of interacting and forming a heterodimer, the respective purified proteins were mixed and the mixture was analysed by native PAGE. The results indicated that the protein complex migrated as a single band in the presence of excess monomeric CFP-10 (Figure S2A). Additionally we used surface plasmon resonance (SPR) assays to corroborate the interaction between the CFP-10 and ESAT-6 proteins. Since the ESAT-6 protein did not bind to the surface of a CM5 sensor chip in the absence of immobilized CFP-10 protein, the results provide supporting evidence for specificity in the interaction between the two recombinant proteins (Figure S2B).

### Isolation of Aptamers against the CFP-10.ESAT-6 Heterodimer

**Figure 2:**
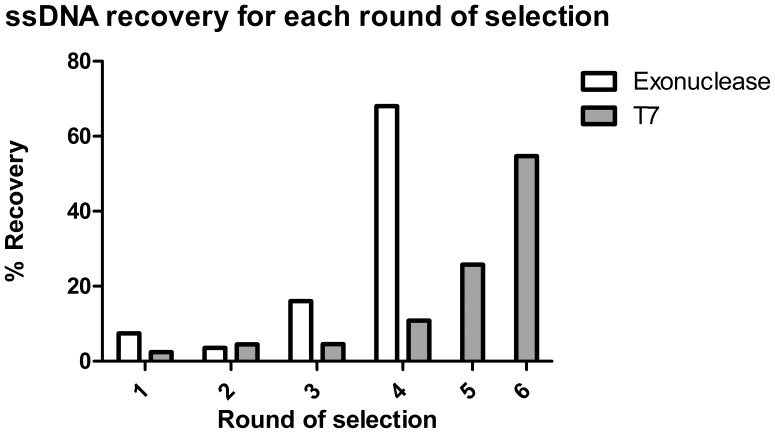
Percentage recovery of ssDNA using different SELEX methods. Both exonuclease- and T7-based methods were used to generate ssDNA, and counter-selection was preformed after round 3 (exonuclease-based SELEX) and 5 (T7-based SELEX).

To isolate aptamers against the CFP-10.ESAT-6 heterodimer, a modified SELEX protocol was followed [35]. The starting library contained a random region of 49 nucleotides, and a complexity of 10^14^ unique sequences was used for the first round of selection. Two methods of selection were used to maximise the number of aptamers that could be isolated. The T7-based SELEX resulted in an enrichment of 54.7% after six rounds of selection and the exonuclease-based SELEX had an enrichment of 68% after four rounds of selection (Figure 2). Counter-selections, to eliminate non-specific binders, were performed after round 3 and 5 for the exonuclease-based and T7-based SELEX, respectively. After selection, the ssDNA pools were purified and cloned. A total of 104 clones were obtained and subjected to nucleotide sequencing. Sequence analysis indicated the presence of 66 unique clones that comprised of five obtained from the exonuclease-based SELEX method and the remainder were obtained using the T7-based SELEX method. Aptamers obtained from both the exonuclease- and the T7-based SELEX methods were used for further studies.

### Binding of ssDNA Aptamers to the CFP-10.ESAT-6 Heterodimer

**Figure 3:**
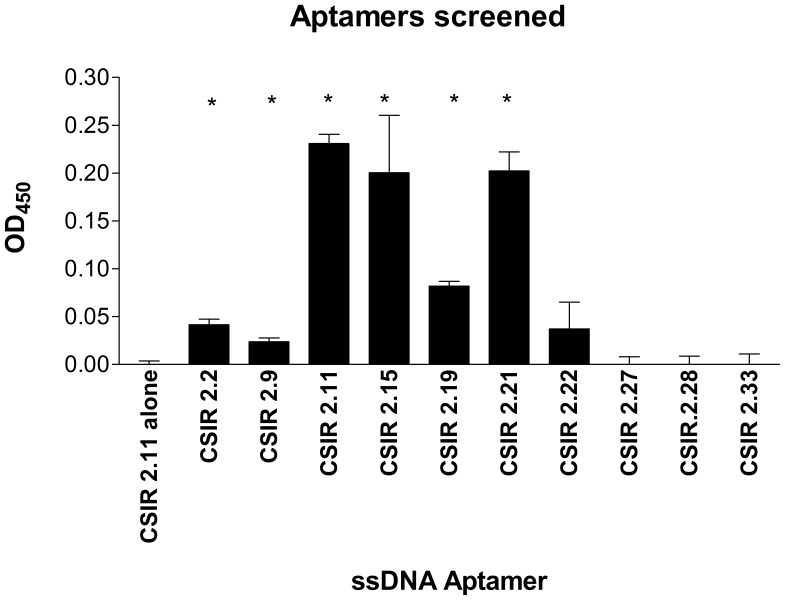
Binding affinity of ssDNA aptamers to the CFP-10.ESAT-6 heterodimer. Results are shown for a selection of biotinylated ssDNA aptamers. Biotinylated ssDNA aptamers that bound significantly (p<0.05) to the heterodimer in an ELONA assay are denoted by the asterisks. Data are presented as means ± standard deviation of the mean.

To determine the binding ability of individual aptamers to the CFP-10.ESAT-6 heterodimer, an ELONA was used in which biotinylated aptamers were added to immobilised heterodimer. Out of the 66 biotinylated ssDNA aptamers screened against the CFP-10.ESAT-6 heterodimer, 24 bound significantly (p<0.05) to the heterodimer. Of these 24 aptamers, six aptamers that displayed the highest OD_450_ readings were chosen for further analysis (Figure 3).

### Aptamer-antibody Competition Binding Assays of Individual ssDNA Aptamers

**Figure 4:**
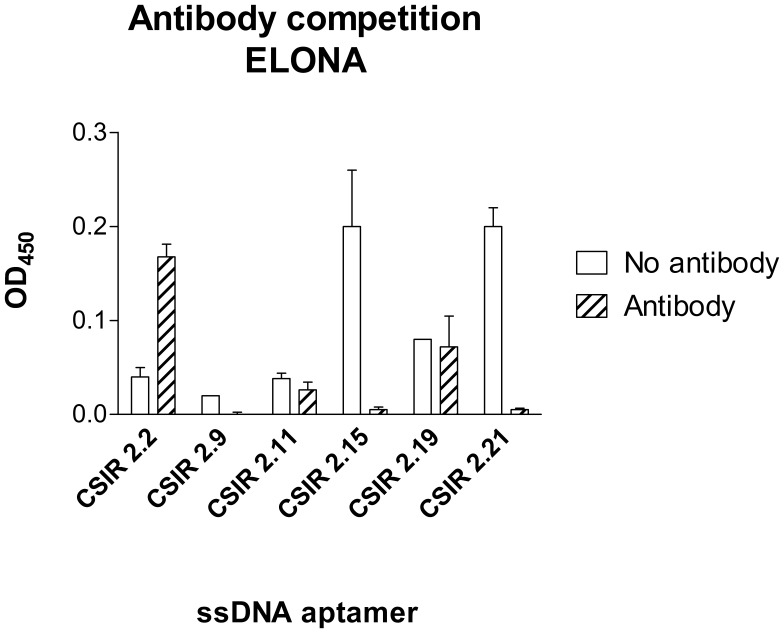
Aptamer-antibody competition binding assay. An ELONA was used to determine binding of selected anti-CFP-10.ESAT-6 biotinylated ssDNA aptamers to the heterodimer in the presence (striped bar) or absence (solid white) of anti-ESAT-6 monoclonal antibody. Data are presented as means ± standard deviations of the mean.

An aptamer-antibody competition assay was performed to determine whether the aptamers could be used in the presence of the anti-ESAT-6 monoclonal antibody. This is important for downstream assays that could involve a sandwich assay using the aptamers. Binding of some ssDNA aptamers such as CSIR 2.9, CSIR 2.15 and CSIR 2.21 was abrogated by the presence of anti-ESAT-6 monoclonal antibody, whereas binding of others such as CSIR 2.11 and CSIR 2.19 was unaffected by the presence of the ESAT-6 monoclonal antibody (Figure 4). This data suggests that in the case of abrogated aptamer binding the presence of the ESAT-6 monoclonal antibody may have caused allosteric effects, thereby blocking binding sites on the heterodimer recognised by the aptamers.

### Binding of Individual ssDNA Aptamers to Protein Monomers

**Figure 5:**
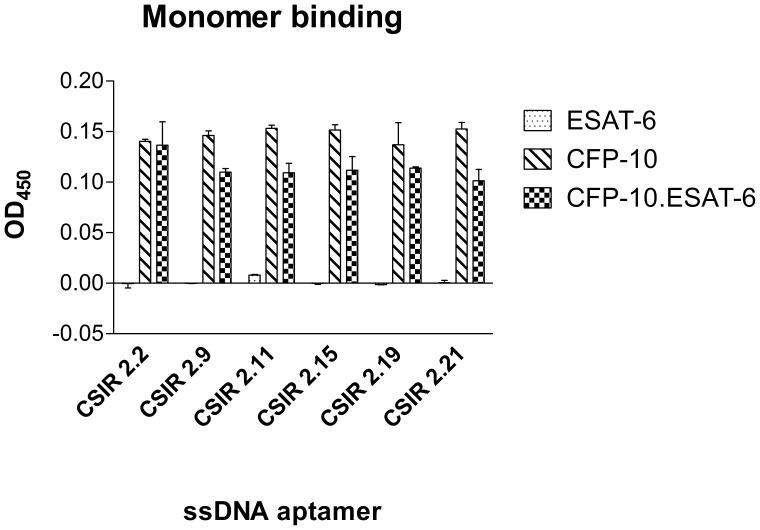
Binding affinity of the selected ssDNA aptamers to CFP-10 and ESAT-6. Binding of the anti-CFP-10.ESAT-6 biotinylated ssDNA aptamers to the ESAT-6 and CFP-10 monomers was determined by an ELONA. Binding of the ssDNA aptamers to the CFP-10.ESAT-6 heterodimer is shown for comparative purposes. Data are presented as means ± standard deviation of the mean.

It was established that the ssDNA aptamers could bind to the CFP-10.ESAT-6 heterodimer; however, it was not yet known whether the aptamers bound preferentially to one of the two monomers (i.e. CFP-10 or ESAT-6) or only bind to the heterodimer. Thus, the binding of the six selected aptamers to CFP-10 and ESAT-6 monomers was determined using ELONA. Interestingly, although the aptamers were isolated against the heterodimer, all six of the selected aptamers recognised the CFP-10 monomer, in addition to the CFP-10.ESAT-6 heterodimer, but none of the aptamers recognised the ESAT-6 monomer (Figure 5).

To confirm that the aptamers could be chemically synthesised and not affect their binding abilities, the six aptamers were synthesised by solid phase with a biotin modification. The binding of these aptamers to the CFP-10.ESAT-6 heterodimer, as well as CFP-10 and ESAT-6 was subsequently repeated. There was no significant (p>0.05) difference in the binding affinity between the *in vitro-* and the solid phase-synthesised aptamers (Figure S3). Both the *in vitro* and chemically-synthesised aptamers showed consistent results in that none of the aptamers recognised the ESAT-6 monomer, while they were able to bind to the CFP-10.ESAT-6 heterodimer and the CFP-10 monomer.

### Dissociation Constant (K_D_) of Individual ssDNA Aptamers

**Figure 6:**
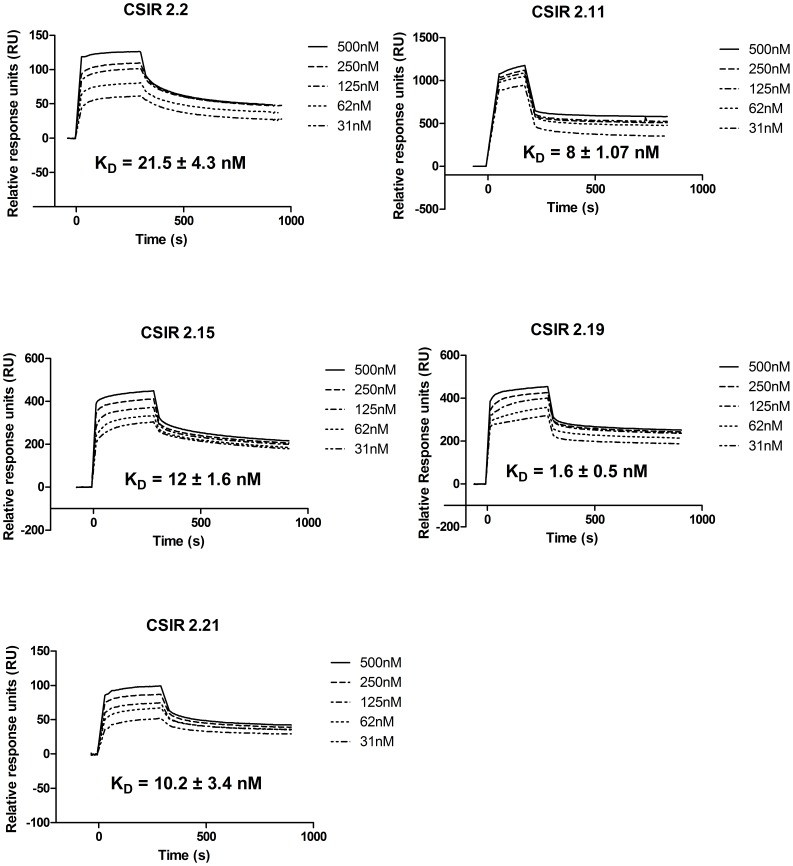
BIAcore sensogram showing the equilibrium dissociation constantss (K_D_) of the selected ssDNA apatmers. The binding kinetics of the ssDNA aptamers to immobilised CFP-10 was determined using BIAcore® 3000 surface plasmon resonance (SPR). In each case the aptamer was iinjected over immobolised CFP-10 with an initial concentration at 500 nM, followed by 250 nM, 125 nM, 62 nM and 31 nM.

To determine the binding kinetics of the aptamers, CFP-10 was amine-coupled onto a CM5 chip, followed by the injection of the aptamers to obtain dissociation equilibrium constant (K_D_) values for each aptamer. All aptamers gave K_D_ values in the lower nanomolar range. CSIR 2.19 had the lowest K_D_ of 1.6±0.5 nM, CSIR 2.11 had a K_D_ of 8±1.07 nM and CSIR 2.2 had comparatively the highest K_D_ of 21.5±4.3 nM (Figure 6). The Chi^2^ values for the kinetic studies ranged between 0.015 and 1.09. Chi^2^ values below 2 illustrate a good fit of data to the 1∶1 Langmuir model; indicating tight affinity of the aptamers to the CFP-10 monomer.

### Effect of Aptamer Folding on Target Binding

CSIR 2.11 was chosen for further studies as it was unaffected in the aptamer-antibody competition assay. For aptamers to be used as detection reagents, they must be stable. Thus, we determined whether a refolding step was necessary for the aptamer to bind to the CFP-10 protein. One batch of CSIR 2.11 was folded as described in the methods and another batch of CSIR 2.11 was used directly after thawing. The results showed that the aptamer can be used directly after thawing without the refolding step (Figure S4). This data is useful for the application of CSIR 2.11 as a detection reagent. It suggests that the aptamer retains a stable structure for prolonged periods without requiring heating and cooling to fold correctly in order for it to bind to CFP-10.

### Specificity Testing

To determine the specificity of the ssDNA aptamers, lysates were prepared from bacteria inhabiting the oral cavity and evaluated for binding to the aptamers in an ELONA. The six tested aptamers had variable binding, although all aptamers were able to recognise *Mycobacterium tuberculosis* lysates and none of the aptamers were able to bind to the lysates of *S. pyogenes* or *C. xerosis* (Table 1, Figure S5). *M. smegmatis, M. bovis* BCG and *S. aureus* lysates were recognised by five of the six aptamers, whereas the *P. aeruginosa* lysate was recognised by three of the six aptamers (Table 1, Figure S5).

**Table 1:** ELONA results of the ssDNA aptamers tested on different bacterial lysates.

Bacterial Lysate	CSIR 2.2	CSIR 2.9	CSIR 2.11	CSIR 2.15	CSIR 2.19	CSIR 2.21
*Mycobacterium tuberculosis*	+	+	+	+	+	+
*Mycobacterium bovis*	+	+	+	+	+	–
*Mycobacterium smegmatis*	+	–	+	+	+	+
*Staphylococcus aureus*	+	+	+	+	+	–
*Pseudomonas aeruginosa*	–	–	+	+	+	–
*Streptococcus pyogenes*	–	–	–	–	–	–
*Corynybacterium xerosis*	–	–	–	–	–	–

+ positive for CFP-10 detection; − negative for CFP-10 detection.

### Antigen Detection in Sputum Samples

To evaluate the aptamers for detecting TB infection using clinical sputum samples, the utility of CSIR 2.11 was tested in 68 sputum samples in an ELONA assay. Based on the ROC curve analysis with a 99% confidence interval, the study showed that in 86% (p<0.0001) of randomly selected cases, a TB-positive individual has a test value (OD_450_) higher than a TB-negative individual. In this study, the cut-point for positive readings based on the ROC curve analysis using the Youden’s index was OD_450_ = 0.2. Using this cut-off, the assay has a specificity of 68.75% and sensitivity of 100% (Table 2, Figure S6). However, when a rule-in disease cut-point is considered the specificity is set at 95% and the sensitivity decreases to 35% (Table 2, Figure S6). This decrease is due to one healthy donor and two TB-negative samples that had high positive readings. As expected, CSIR 3.13, which was used as a negative control, did not detect TB in the sputum samples (Figure S7). The coefficient of variance was ranged between 2 and 10%, indicating the repeatability of the test. A more specific aptamer, CSIR 2.21, was compared to CSIR 2.11 using 28 sputum samples. The data shows that the two aptamers were comparable with a specificity of 85% and sensitivity of 10% using a rule-in disease cut-point, and a specificity of 60% and sensitivity of 65% using the Youden’s index (Figure S8).

**Table 2:** ELONA results of the ssDNA aptamer CSIR 2.11 tested as a detection reagent for active TB in sputum samples.

Tuberculosis status	Youden’s index*				Rule-in disease cut-point	
	Subjects (n)	Positive forCFP-10		Sensitivity	Positive forCFP-10	Sensitivity
**Sputum samples positive for TB**	**20**	**20**	**100%**		**7**	**35%**
Smear positive, culture positive	15	15			6	
Smear negative, culture positive	5	5			1	
			**Specificity**			**Specificity**
**Sputum samples negative for TB**	**20**	**8**	**68.75%**		**2**	**95%**
Latent infection	5	2			1	
Smear negative, culture negative	15	6			1	
					
**Healthy laboratory volunteers negative for TB** 1	**28**	**7**			**2**	

1 The two donors who gave a single sample were not detected as TB positive in the ELONA. Of the 13 donors that gave two sputum samples, two cases had both samples as positive and in three of the cases only the second sample gave a positive reading.

* Youden’s index is one that selects the best trade-off between sensitivity and specificity using a ROC curve, and may not correspond to a cut-point that rules in or rules out disease.

## Discussion

We isolated aptamers against the CFP-10.ESAT-6 heterodimer to be used as potential TB detection reagents. In a small case controlled study, the aptamer-based assay had a sensitivity of 100% and a specificity of 68.75% using Youden’s index, which is encouraging for development of the aptamers as detection reagents for active TB.

It was interesting to note that even though the aptamers were raised against the heterodimer, all aptamers displayed a preference for one of the monomers, namely CFP-10, over the heterodimer. This is a unique situation and had not been previously observed when RNA aptamers were raised against the human interleukin-17A/F heterodimer [39]. This could be due to RNA aptamers forming more stable secondary and tertiary structures than ssDNA aptamers. Alternatively, the ESAT-6 binding sites may not be accessible to the aptamers when ESAT-6 is bound to CFP-10. Aptamers are known to bind preferentially to functional parts of proteins [40], [41], [42], [43]. The flexible C terminus of CFP-10 has been shown to be involved in interactions with cell surfaces. Indeed, deletion of the 87 amino acids at the C terminal of CFP-10 inhibited binding of the protein complex to the cell surface, whereas a similar deletion in ESAT-6 had no effect [16]. This suggests that the C terminus of CFP-10 constitutes the functional part of the heterodimer and could therefore explain the preferential binding of the aptamers to CFP-10. In this study characterisation of the aptamers revealed that the aptamers had a tight affinity to CFP-10, with the tested aptamers having dissociation constants in the low nanomolar range.

This study used oral cavity bacteria to determine the specificity of the aptamers. Oral cavity bacteria was chosen due to the wide diversity of bacteria present that includes gram-positive bacteria, gram-negative bacteria, anaerobic bacteria, mycobacteria and fungal species. Lower respiratory tract bacteria are present at much lower concentrations than oral cavity bacteria and require the use of specialised techniques such as bronchoscopy [44]. The bacterial lysate study revealed that the aptamers were able to recognise *S. aureus, M. smegmatis*, *M. bovis* BCG and, in some cases, *P. aeruginosa*. This may be due to the fact that *M. smegmatis* secretes the CFP-10 protein [45], and although the RD1 region has been removed from *M. bovis* BCG, the mycobacterium genome has 23 ESAT-6 homologues (such as CFP-10, Esx G and Esx H). Thus, the aptamers might recognise one of theESAT-6-like proteins that are encoded by one of the other 10 genomic loci [46]. ESAT-6-like homologues have also been described in the genomes of other gram-positive bacteria like *Bacillus subtilis, B. anthracis, Clostridium acetobutylicum, Listeria monocytogenes*, and *S. aureus* [47], [48]. The presence of these ESAT-6-like homologues in *S. aureus* may explain why aptamers raised against the CFP-10.ESAT-6 heterodimer also bind to lysates of *S. aureus*.

Despite binding of the aptamer to *M. smegmatis*, *M. bovis* BCG and *S. aureus*, CSIR 2.11 was selected and tested for its ability to detect TB using sputum samples. CSIR 2.11 had a specificity of 68.75% using Youden’s index, which is lower than that observed for antibodies raised to detect the CFP-10 monomer [20]. The sensitivity and specificity of a more specific aptamer, CSIR 2.21, was not significantly different from that of CSIR 2.11 in a comparative study using 28 sputum samples. Possible reasons for some of the false positive readings in this study might be as a result of *S. aureus* co-infection [47] or from misclassification bias due to subclinical TB; however, the patients did not return to the hospital and thus no further follow up could be done to clarify this point. Additionally, the lowered specificity may be attributed to antigen persistence in patients with previous TB infection [49]. Alternatively, long-term persister organisms could be secreting the culture filtrate proteins at very low levels, detectable by the aptamers but not by antibodies.

An encouraging aspect of the aptamer-based diagnostic assay, in this preliminary study, is that it performed reasonably well in both HIV-infected and HIV-uninfected patients. Further studies, after appropriate optimisation, will be required in HIV-infected individuals. Diagnostic tests may vary in their performance in HIV-infected individuals. GeneXpert® has sub-optimal negative predictive values in HIV-infected individuals [11], whilst the urine LAM-assay performs optimally in HIV-infected individuals with low CD4 counts [50], [51]. The sensitivity of the aptamer-based test is comparable to that of an antibody-based ELISA in a recent study by Feng *et al.* [20], with both studies having sensitivities of over 80% for the detection of CFP-10 in clinical specimens. Compared to the antibody-based assays, aptamer-based assays have greater stability under a wide range of conditions, and can be repeatedly used without losing their binding ability. Aptamers are, therefore, a promising class of detection reagents for the development of improved diagnostics [52]. Moreover, this study, as well as previous studies have shown that aptamers can be chemically synthesised, which allows for their rapid production in large quantities with excellent reproducibility of results [27], [52], [53].

Limitations of the current aptamer-based assay are the lack of a rapid readout, as ELONA takes several hours to complete, as well as antigenic cross-reactivity. The cross-reactivity of the aptamer-based assay may be improved through optimisation of the aptamers, which may include truncation of the current 90-mer parent aptamers to minimum sequences essential for binding to the target, which in some cases improves the kinetics of the aptamers [54]. By truncating the aptamers not only will the cost of production be reduced, but it may also prevent non-specific binding. Furthermore, a sandwich assay can be developed that makes use of two aptamers; one as a capture and the other as a detector molecule. By making use of such a sandwich assay the specificity of either the capture or the detector aptamer can be lower, provided that the combination of the two is highly specific to the antigen in question.

This study showed that it is feasible to develop aptamers to relatively TB-specific antigens and thereby detect these antigens in human biological samples obtained from patients with and without active TB. However, further optimisation is required to improve performance outcomes, followed by validation in larger appropriately designed studies using biological samples from different body compartments. This will lay the foundation for their inclusion and evaluation in point-of-care detection platforms.

## Supporting Information

Figure S1 **Expression and purification of CFP-10 and ESAT-6.** (A) SDS analysis of purified CFP-10. Lane 1, Precision Plus Protein™ Kaleidoscope Standards (BioRad); and lane 2, eluate from Ni-NTA column indicating the presence of pure monomeric CFP-10. (B) Immunoblot analysis of the purified CFP-10 protein, indicating that the anti-CFP-10 polyclonal antibody reacted specifically with CFP-10 (lane 1), but not with ESAT-6 (negative control, lane 2). (C) SDS-PAGE analysis of purified ESAT-6. Lane 1, Precision Plus Protein™ Kaleidoscope Standards (BioRad); and lane 2, eluate from Ni-NTA column indicating the presence of pure monomeric ESTA-6. (D) Immunoblot analysis of the purified ESAT-6 protein, indicating that the anti-ESAT-6 monoclonal antibody reacted specifically with ESAT-6 (lane 1), but not with CFP-10 (negative control, lane 2).
(TIF)

Figure S2 **Complex formation by purified recombinant CFP-10 and ESAT-6 proteins.** (A) Native polyacrylamide gel of purified recombinant ESAT-6 (lane 1) and CFP-10 (lane 2) proteins, and a mixture of the individual proteins (lane 3). (B) Confirmation of CFP-10.ESAT-6 complex formation by surface plasmon resonance (SPR). ESAT-6 was injected on a CFP-10 surface from t_0_ to t_600_, followed by removal of unbound ESAT-6 protein. A control experiment was likewise performed on a CM5 sensor chip devoid of CFP-10.
(TIF)

Figure S3 **Binding affinity of solid phase-synthesised ssDNA aptamers.** In an ELONA the selected aptamers showed binding to CFP-10 and CFP-10.ESAT-6, but not to ESAT-6. These results are in agreement with those presented in Fig. 5, indicating that chemical synthesis of the aptamers does not influence their binding ability. Data are presented as means ± standard deviations of the mean.
(TIF)

Figure S4 **Binding of folded** **and unfolded aptamer CSIR 2.11 to CFP-10.** One batch of CSIR 2.11 was refolded and a second batch was used directly after thawing in the ELONA. No significant differences were observed in the binding capabilities of the folded and the unfolded aptamers, indicating that a refolding step is not necessary for binding of the aptamer to the target protein. Data are presented as means ± standard deviation of the mean.
(TIF)

Figure S5 **Binding of selected ssDNA aptamers to lysates of oral cavity bacteria.** An ELONA was used to test binding of the selected ssDNA aptamers to lysates prepared from *M. tuberculosis*, *M. smegmatis*, *M. bovis BCG, P. aeruginosa*, *S. aureus*, *C. xerosis* and *S. pyogenes*.
(TIF)

Figure S6 **Evaluation of sputum samples using CSIR 2.11** **as a** **detection reagent.** The aptamer was tested on three groups of samples (A) Definite TB, (B) Latent TB and TB negative and (C) healthy laboratory volunteers. Using Youden’s index, the cut-point for positive samples was set at an OD_450_ of 0.2 and is indicated by the dotted line. Using the rule-in disease, the cut-point for positive samples is an OD_450_ of 0.8 and is demarcated by the solid line. Data are presented as means ± standard deviation of the mean.
(TIF)

Figure S7 **Evaluation of sputum samples using a control aptamer CSIR 3.13 as a detection reagent.** Controls included were an aptamer-alone control, a sputum-alone control, a positive control (CFP-10 plus CSIR 2.11), as well as sputum samples that were detected by a ssDNA aptamer (CSIR 3.13) that was selected using the same library but against a different target. Data are represented as means ± standard deviation of the means.
(TIF)

Figure S8 **Comparison of CSIR 2.11 and CSIR 2.21 using sputum samples.** The sensitivity and specificity of CSIR 2.11 and a more specific aptamer, CSIR 2.21, were compared using 28 sputum samples. Using Youden’s index, the cut-point for positive samples was set at an OD_450_ of 0.1 and is indicated by the solid line. Using the rule-in disease, the cut-point for positive samples is an OD_450_ of 0.62 and is demarcated by the dotted line. Data are presented as means ± standard deviation of the mean. Samples that gave a positive result when using CSIR 2.21as a detection molecule are denoted as CSIR 2.21 positive, while negative samples are denoted as CSIR 2.21 negative. Samples that gave a positive result when using CSIR 2.11 as a detection molecule are denoted as CSIR 2.11 positive, while negative samples are denoted CSIR 2.11 negative.
(TIF)

## References

[1] WHO (2011) WHO report 2011: Global Tuberculosis control. World Health Organization.

[2] Gandhi NR, Nunn P, Dheda K, Schaaf HS, Zignol M, et al (2010) Multidrug-resistant and extensively drug-resistant tuberculosis: a threat to global control of tuberculosis. Lancet 375: 1830–1843. 20488523 10.1016/S0140-6736(10)60410-2

[3] Jarand J, Shean K, O'Donnell M, Loveday M, Kvasnovsky C, et al (2010) Extensively drug-resistant tuberculosis (XDR-TB) among health care workers in South Africa. Trop Med Int Health 15: 1179–1184. 20831672 10.1111/j.1365-3156.2010.02590.x

[4] O'Donnell MR, Jarand J, Loveday M, Padayatchi N, Zelnick J, et al (2010) High incidence of hospital admissions with multidrug-resistant and extensively drug-resistant tuberculosis among South African health care workers. Ann Intern Med 153: 516–522. 20956708 10.1059/0003-4819-153-8-201010190-00008 PMC3074259

[5] Alderson MR, Bement T, Day CH, Zhu L, Molesh D, et al (2000) Expression cloning of an immunodominant family of Mycobacterium tuberculosis antigens using human CD4(+) T cells. J Exp Med 191: 551–560. 10662800 10.1084/jem.191.3.551 PMC2195809

[6] Steingart KR, Henry M, Ng V, Hopewell PC, Ramsay A, et al (2006) Fluorescence versus conventional sputum smear microscopy for tuberculosis: a systematic review. Lancet Infect Dis 6: 570–581. 16931408 10.1016/S1473-3099(06)70578-3

[7] Steingart KR, Ng V, Henry M, Hopewell PC, Ramsay A, et al (2006) Sputum processing methods to improve the sensitivity of smear microscopy for tuberculosis: a systematic review. Lancet Infect Dis 6: 664–674. 17008175 10.1016/S1473-3099(06)70602-8

[8] Andersen P, Munk ME, Pollock JM, Doherty TM (2000) Specific immune-based diagnosis of tuberculosis. Lancet 356: 1099–1104. 11009160 10.1016/s0140-6736(00)02742-2

[9] Dheda K, Davids V, Lenders L, Roberts T, Meldau R, et al (2010) Clinical utility of a commercial LAM-ELISA assay for TB diagnosis in HIV-infected patients using urine and sputum samples. PLoS One 5: e9848. 20352098 10.1371/journal.pone.0009848 PMC2844421

[10] Dheda K, Van-Zyl Smit RN, Sechi LA, Badri M, Meldau R, et al (2009) Clinical diagnostic utility of IP-10 and LAM antigen levels for the diagnosis of tuberculous pleural effusions in a high burden setting. PLoS One 4: e4689. 19277111 10.1371/journal.pone.0004689 PMC2650091

[11] Boehme CC, Nabeta P, Hillemann D, Nicol MP, Shenai S, et al (2010) Rapid molecular detection of tuberculosis and rifampin resistance. N Engl J Med 363: 1005–1015. 20825313 10.1056/NEJMoa0907847 PMC2947799

[12] Arend SM, Andersen P, van Meijgaarden KE, Skjot RL, Subronto YW, et al (2000) Detection of active tuberculosis infection by T cell responses to early-secreted antigenic target 6-kDa protein and culture filtrate protein 10. J Infect Dis 181: 1850–1854. 10823800 10.1086/315448

[13] Brodin P, de Jonge MI, Majlessi L, Leclerc C, Nilges M, et al (2005) Functional analysis of early secreted antigenic target-6, the dominant T-cell antigen of Mycobacterium tuberculosis, reveals key residues involved in secretion, complex formation, virulence, and immunogenicity. J Biol Chem 280: 33953–33959. 16048998 10.1074/jbc.M503515200

[14] Dillon DC, Alderson MR, Day CH, Bement T, Campos-Neto A, et al (2000) Molecular and immunological characterization of Mycobacterium tuberculosis CFP-10, an immunodiagnostic antigen missing in Mycobacterium bovis BCG. J Clin Microbiol 38: 3285–3290. 10970372 10.1128/jcm.38.9.3285-3290.2000 PMC87375

[15] Kulshrestha A, Gupta A, Verma N, Sharma SK, Tyagi AK, et al (2005) Expression and purification of recombinant antigens of Mycobacterium tuberculosis for application in serodiagnosis. Protein Expr Purif 44: 75–85. 15982900 10.1016/j.pep.2005.04.014

[16] Renshaw PS, Lightbody KL, Veverka V, Muskett FW, Kelly G, et al (2005) Structure and function of the complex formed by the tuberculosis virulence factors CFP-10 and ESAT-6. Embo J 24: 2491–2498. 15973432 10.1038/sj.emboj.7600732 PMC1176459

[17] Renshaw PS, Panagiotidou P, Whelan A, Gordon SV, Hewinson RG, et al (2002) Conclusive evidence that the major T-cell antigens of the Mycobacterium tuberculosis complex ESAT-6 and CFP-10 form a tight, 1:1 complex and characterization of the structural properties of ESAT-6, CFP-10, and the ESAT-6*CFP-10 complex. Implications for pathogenesis and virulence. J Biol Chem 277: 21598–21603. 11940590 10.1074/jbc.M201625200

[18] Andersen P (1994) The T cell response to secreted antigens of Mycobacterium tuberculosis. Immunobiology 191: 537–547. 7713568 10.1016/S0171-2985(11)80460-2

[19] Skjot RL, Oettinger T, Rosenkrands I, Ravn P, Brock I, et al (2000) Comparative evaluation of low-molecular-mass proteins from Mycobacterium tuberculosis identifies members of the ESAT-6 family as immunodominant T-cell antigens. Infect Immun 68: 214–220. 10603390 10.1128/iai.68.1.214-220.2000 PMC97123

[20] Feng TT, Shou CM, Shen L, Qian Y, Wu ZG, et al (2011) Novel monoclonal antibodies to ESAT-6 and CFP-10 antigens for ELISA-based diagnosis of pleural tuberculosis. Int J Tuberc Lung Dis 15: 804–810. 21575303 10.5588/ijtld.10.0393

[21] Mabey D, Peeling RW, Ustianowski A, Perkins MD (2004) Diagnostics for the developing world. Nat Rev Microbiol 2: 231–240. 15083158 10.1038/nrmicro841

[22] Ellington AD, Szostak JW (1990) In vitro selection of RNA molecules that bind specific ligands. Nature 346: 818–822. 1697402 10.1038/346818a0

[23] Robertson DL, Joyce GF (1990) Selection in vitro of an RNA enzyme that specifically cleaves single-stranded DNA. Nature 344: 467–468. 1690861 10.1038/344467a0

[24] Tuerk C, Gold L (1990) Systematic evolution of ligands by exponential enrichment: RNA ligands to bacteriophage T4 DNA polymerase. Science 249: 505–510. 2200121 10.1126/science.2200121

[25] Low SY, Hill JE, Peccia J (2009) DNA aptamers bind specifically and selectively to (1–>3)-beta-D-glucans. Biochem Biophys Res Commun 378: 701–705. 19061867 10.1016/j.bbrc.2008.11.087 PMC2638985

[26] Lee JF, Stovall GM, Ellington AD (2006) Aptamer therapeutics advance. Curr Opin Chem Biol 10: 282–289. 16621675 10.1016/j.cbpa.2006.03.015

[27] Liu J, Lu Y (2005) Fast colorimetric sensing of adenosine and cocaine based on a general sensor design involving aptamers and nanoparticles. Angew Chem Int Ed Engl 45: 90–94. 16292781 10.1002/anie.200502589

[28] Liu J, Lu Y (2006) Preparation of aptamer-linked gold nanoparticle purple aggregates for colorimetric sensing of analytes. Nat Protoc 1: 246–252. 17406240 10.1038/nprot.2006.38

[29] Wang JY, Chou CH, Lee LN, Hsu HL, Jan IS, et al (2007) Diagnosis of tuberculosis by an enzyme-linked immunospot assay for interferon-gamma. Emerg Infect Dis 13: 553–558. 17553269 10.3201/eid1304.051195 PMC2725949

[30] Wang L, Liu X, Hu X, Song S, Fan C (2006) Unmodified gold nanoparticles as a colorimetric probe for potassium DNA aptamers. Chem Commun (Camb): 3780–3782. 10.1039/b607448k 16969455

[31] Wei H, Li B, Li J, Wang E, Dong S (2007) Simple and sensitive aptamer-based colorimetric sensing of protein using unmodified gold nanoparticle probes. Chem Commun (Camb): 3735–3737. 10.1039/b707642h 17851611

[32] Shum KT, Lui EL, Wong SC, Yeung P, Sam L, et al (2011) Aptamer-Mediated Inhibition of Mycobacterium tuberculosis Polyphosphate Kinase 2. Biochemistry 50: 3261–3271. 21381755 10.1021/bi2001455

[33] Chen F, Zhou J, Luo F, Mohammed AB, Zhang XL (2007) Aptamer from whole-bacterium SELEX as new therapeutic reagent against virulent Mycobacterium tuberculosis. Biochem Biophys Res Commun 357: 743–748. 17442275 10.1016/j.bbrc.2007.04.007

[34] Qin L, Zheng R, Ma Z, Feng Y, Liu Z, et al (2009) The selection and application of ssDNA aptamers against MPT64 protein in Mycobacterium tuberculosis. Clin Chem Lab Med 47: 405–411. 19284297 10.1515/CCLM.2009.097

[35] Jhaveri SD, Ellington AD (2001) In vitro selection of RNA aptamers to a protein target by filter immobilization. Curr Protoc Nucleic Acid Chem Chapter 9: Unit 9 3. 10.1002/0471142700.nc0903s00 18428881

[36] Avci-Adali M, Paul A, Wilhelm N, Ziemer G, Wendel HP (2010) Upgrading SELEX technology by using lambda exonuclease digestion for single-stranded DNA generation. Molecules 15: 1–11. 10.3390/molecules15010001 PMC6256929 20110867

[37] Drolet DW, Moon-McDermott L, Romig TS (1996) An enzyme-linked oligonucleotide assay. Nat Biotechnol 14: 1021–1025. 9631044 10.1038/nbt0896-1021

[38] Chaudhary VK, Kulshreshta A, Gupta G, Verma N, Kumari S, et al (2005) Expression and purification of recombinant 38-kDa and Mtb81 antigens of Mycobacterium tuberculosis for application in serodiagnosis. Protein Expr Purif 40: 169–176. 15721785 10.1016/j.pep.2004.10.016

[39] Adachi H, Ishiguro A, Hamada M, Sakota E, Asai K, et al (2011) Antagonistic RNA aptamer specific to a heterodimeric form of human interleukin-17A/F. Biochimie 93: 1081–1088. 21524680 10.1016/j.biochi.2011.04.003

[40] Huang DB, Vu D, Cassiday LA, Zimmerman JM, Maher LJ, 3rd, et al (2003) Crystal structure of NF-kappaB (p50)2 complexed to a high-affinity RNA aptamer. Proc Natl Acad Sci U S A 100: 9268–9273. 12886018 10.1073/pnas.1632011100 PMC170907

[41] Jaeger J, Restle T, Steitz TA (1998) The structure of HIV-1 reverse transcriptase complexed with an RNA pseudoknot inhibitor. Embo J 17: 4535–4542. 9687519 10.1093/emboj/17.15.4535 PMC1170784

[42] Kettenberger H, Eisenfuhr A, Brueckner F, Theis M, Famulok M, et al (2006) Structure of an RNA polymerase II-RNA inhibitor complex elucidates transcription regulation by noncoding RNAs. Nat Struct Mol Biol 13: 44–48. 16341226 10.1038/nsmb1032

[43] Storz G, Altuvia S, Wassarman KM (2005) An abundance of RNA regulators. Annu Rev Biochem 74: 199–217. 15952886 10.1146/annurev.biochem.74.082803.133136

[44] Charlson ES, Bittinger K, Haas AR, Fitzgerald AS, Frank I, et al (2011) Topographical continuity of bacterial populations in the healthy human respiratory tract. Am J Respir Crit Care Med 184: 957–963. 21680950 10.1164/rccm.201104-0655OC PMC3208663

[45] Converse SE, Cox JS (2005) A protein secretion pathway critical for Mycobacterium tuberculosis virulence is conserved and functional in Mycobacterium smegmatis. J Bacteriol 187: 1238–1245. 15687187 10.1128/JB.187.4.1238-1245.2005 PMC545616

[46] Maciag A, Piazza A, Riccardi G, Milano A (2009) Transcriptional analysis of ESAT-6 cluster 3 in Mycobacterium smegmatis. BMC Microbiol 9: 48. 19257911 10.1186/1471-2180-9-48 PMC2660348

[47] Gey Van Pittius NC, Gamieldien J, Hide W, Brown GD, Siezen RJ, et al (2001) The ESAT-6 gene cluster of Mycobacterium tuberculosis and other high G+C Gram-positive bacteria. Genome Biol 2: RESEARCH0044. 11597336 10.1186/gb-2001-2-10-research0044 PMC57799

[48] Pallen M, Chaudhuri R, Khan A (2002) Bacterial FHA domains: neglected players in the phospho-threonine signalling game? Trends Microbiol 10: 556–563. 12564991 10.1016/s0966-842x(02)02476-9

[49] Gomez JE, McKinney JD (2004) M. tuberculosis persistence, latency, and drug tolerance. Tuberculosis (Edinb) 84: 29–44. 14670344 10.1016/j.tube.2003.08.003

[50] Peter J, Green C, Hoelscher M, Mwaba P, Zumla A, et al (2010) Urine for the diagnosis of tuberculosis: current approaches, clinical applicability, and new developments. Curr Opin Pulm Med 16: 262–270. 20375787 10.1097/MCP.0b013e328337f23a PMC5454484

[51] Peter JG, Theron G, van Zyl-Smit R, Haripersad A, Mottay L, et al. (2012) Diagnostic accuracy of a urine LAM strip-test for TB detection in HIV-infected hospitalised patients. Eur Respir J. 10.1183/09031936.00201711 PMC5523653 22362849

[52] Yang CJ, Jockusch S, Vicens M, Turro NJ, Tan W (2005) Light-switching excimer probes for rapid protein monitoring in complex biological fluids. Proc Natl Acad Sci U S A 102: 17278–17283. 16301535 10.1073/pnas.0508821102 PMC1297691

[53] Laurenson S, Pett MR, Hoppe-Seyler K, Denk C, Hoppe-Seyler F, et al (2011) Development of peptide aptamer microarrays for detection of HPV16 oncoproteins in cell extracts. Anal Biochem 410: 161–170. 21059336 10.1016/j.ab.2010.10.038

[54] Shangguan D, Tang Z, Mallikaratchy P, Xiao Z, Tan W (2007) Optimization and modifications of aptamers selected from live cancer cell lines. Chembiochem 8: 603–606. 17373017 10.1002/cbic.200600532

