# Supplementary figures and images for: Selection and Application of ssDNA Aptamers to Detect Active TB from Sputum Samples
Source: PLoS One. 2012 Oct 4;7(10):e46862. doi: 10.1371/journal.pone.0046862 (PMC3464247; doi:10.1371/journal.pone.0046862)

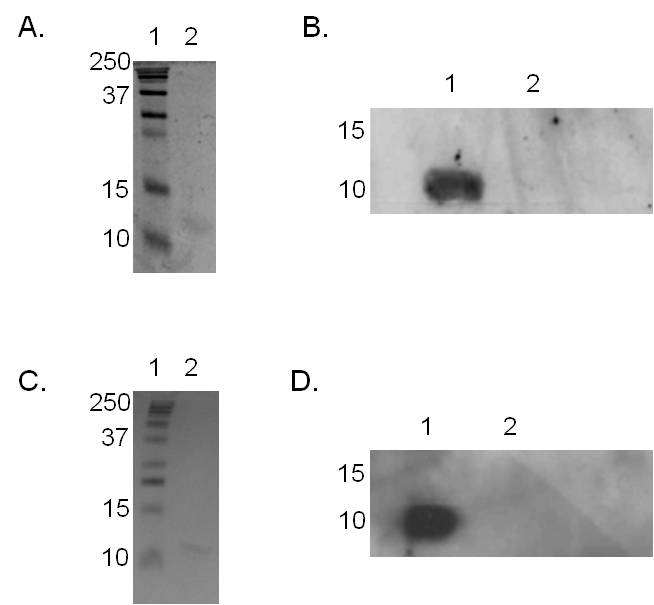

Supplement: Figure S1 — Expression and purification of CFP-10 and ESAT-6. (A) SDS analysis of purified CFP-10. Lane 1, Precision Plus Protein™ Kaleidoscope Standards (BioRad); and lane 2, eluate from Ni-NTA column indicating the presence of pure monomeric CFP-10. (B) Immunoblot analysis of the purified CFP-10 protein, indicating that the anti-CFP-10 polyclonal antibody reacted specifically with CFP-10 (lane 1), but not with ESAT-6 (negative control, lane 2). (C) SDS-PAGE analysis of purified ESAT-6. Lane 1, Precision Plus Protein™ Kaleidoscope Standards (BioRad); and lane 2, eluate from Ni-NTA column indicating the presence of pure monomeric ESTA-6. (D) Immunoblot analysis of the purified ESAT-6 protein, indicating that the anti-ESAT-6 monoclonal antibody reacted specifically with ESAT-6 (lane 1), but not with CFP-10 (negative control, lane 2). (TIF) [file pone.0046862.s001.tif]

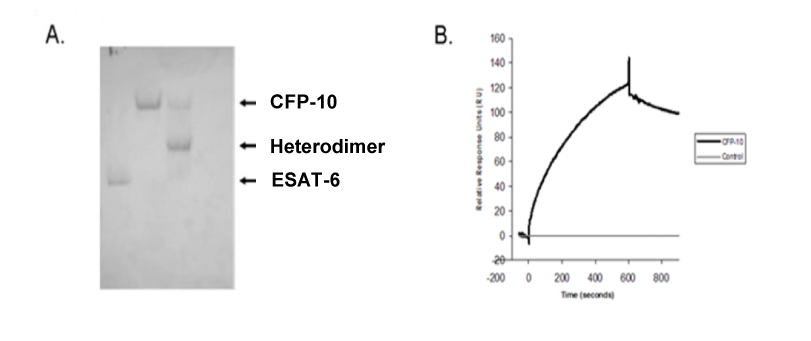

Supplement: Figure S2 — Complex formation by purified recombinant CFP-10 and ESAT-6 proteins. (A) Native polyacrylamide gel of purified recombinant ESAT-6 (lane 1) and CFP-10 (lane 2) proteins, and a mixture of the individual proteins (lane 3). (B) Confirmation of CFP-10.ESAT-6 complex formation by surface plasmon resonance (SPR). ESAT-6 was injected on a CFP-10 surface from t0 to t600, followed by removal of unbound ESAT-6 protein. A control experiment was likewise performed on a CM5 sensor chip devoid of CFP-10. (TIF) [file pone.0046862.s002.tif]

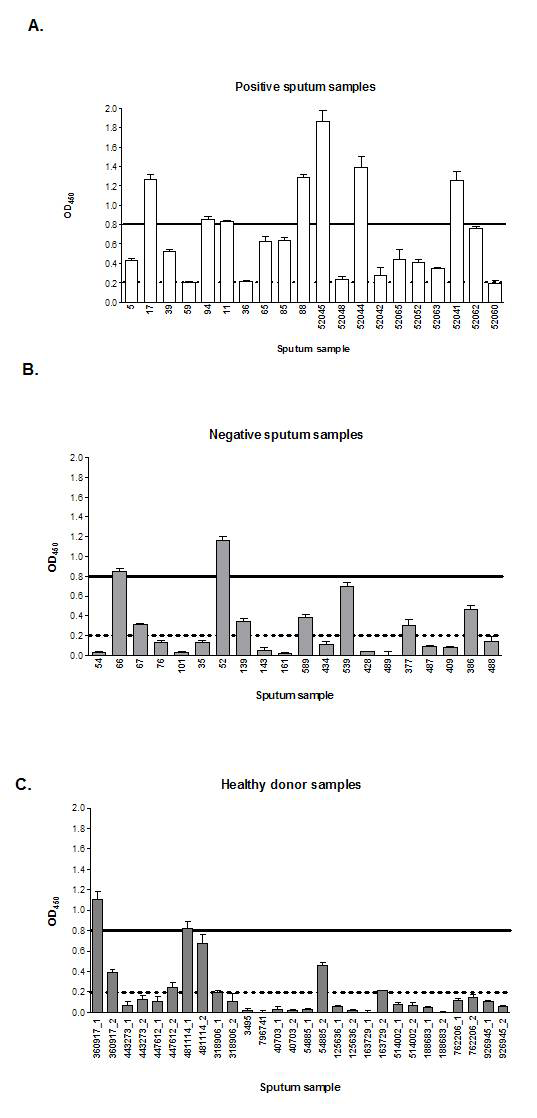

Supplement: Figure S6 — Evaluation of sputum samples using CSIR 2.11 as a detection reagent. The aptamer was tested on three groups of samples (A) Definite TB, (B) Latent TB and TB negative and (C) healthy laboratory volunteers. Using Youden’s index, the cut-point for positive samples was set at an OD450 of 0.2 and is indicated by the dotted line. Using the rule-in disease, the cut-point for positive samples is an OD450 of 0.8 and is demarcated by the solid line. Data are presented as means ± standard deviation of the mean. (TIF) [file pone.0046862.s006.tif]
